# Evolution in Long-Term Stationary-Phase Batch Culture: Emergence of Divergent Escherichia coli Lineages over 1,200 Days

**DOI:** 10.1128/mBio.03337-20

**Published:** 2021-01-26

**Authors:** Nicole R. Ratib, Fabian Seidl, Ian M. Ehrenreich, Steven E. Finkel

**Affiliations:** a Molecular and Computational Biology Section, Department of Biological Sciences, University of Southern California, Los Angeles, California, USA; University of Oklahoma Health Sciences Center

**Keywords:** bacterial evolution, bacterial survival, long-term stationary phase

## Abstract

In natural environments, bacteria survive conditions of starvation and stress. Long-term batch cultures are an excellent laboratory system to study adaptation during nutrient stress because cells can incubate for months to years without the addition of nutrients. During long-term batch culture, cells adapt to acquire energy from cellular detritus, creating a complex and dynamic environment for mutants of increased relative fitness to exploit. Here, we analyzed the genomes of 1,117 clones isolated from a single long-term batch culture incubated for 1,200 days. A total of 679 mutations included single nucleotide polymorphisms, indels, mobile genetic element movement, large deletions up to 64 kbp, and amplifications up to ∼500 kbp. During the 3.3-year incubation, two main lineages diverged, evolving continuously. At least twice, a previously fixed mutation reverted back to the wild-type allele, suggesting beneficial mutations may later become maladaptive due to the dynamic environment and changing selective pressures. Most of the mutated genes encode proteins involved in metabolism, transport, or transcriptional regulation. Clones from the two lineages are physiologically distinct, based on outgrowth in fresh medium and competition against the parental strain. Similar population dynamics and mutations in *hfq*, *rpoS*, *paaX*, *lrp*, *sdhB*, and *dtpA* were detected in three additional parallel populations sequenced through day 60, providing evidence for positive selection. These data provide new insight into the population structure and mutations that may be beneficial during periods of starvation in evolving bacterial communities.

## Abstract (precis)

Bacteria have remarkable metabolic capabilities and adaptive plasticity, enabling them to survive in changing environments. In nature, bacteria spend a majority of their time in a state of slow growth or maintenance, scavenging nutrients for survival.

## INTRODUCTION

Bacteria in the natural world must compete for finite resources, which eventually limit their growth, causing bacteria to enter a state of prolonged starvation (1). When nutrients are again available, bacteria will resume dividing exponentially, but eventually cells enter another state of prolonged starvation, creating a cycle of “feast and famine.” To study microbial evolution, investigators use several experimental platforms, including serial passage of bacterial populations (2, 3) or maintaining populations in chemostats (4), providing valuable insights into adaptation, particularly during exponential growth and early stationary phase. What is lacking, however, is similar detailed analyses of the population dynamics and potential mechanisms bacteria employ to survive during prolonged starvation in an environment where cells must scavenge nutrients for survival.

The long-term batch culture system provides a robust platform for studying adaptation of bacteria, such as Escherichia coli, during prolonged nutrient stress (5). Long-term batch cultures are initiated by inoculating bacteria at low cell density into a rich medium, such as lysogeny broth/Luria-Bertani broth (LB), where cells undergo the well-characterized first three phases of growth in the laboratory: lag phase, log or exponential phase, and stationary phase (6). Following stationary phase, viable cell counts decrease during death phase, where, depending on the strain, growth medium, and growth vessel, approximately 99% of cells die (7, 8). After death phase, the surviving population enters long-term stationary phase (LTSP), where cells can survive for years without the addition of nutrients (5). Since no new nutrients are added to long-term batch cultures, survival during LTSP requires cells to obtain energy and nutrients from the detritus of dead cells, such as amino acids or nucleic acids (9). Characterization of previously isolated LTSP mutants has revealed an increased ability to utilize single amino acids as a sole carbon source in all mutants (9). Microbes in nature likely experience similar selective pressures to those in LTSP, scavenging nutrients to survive periods of starvation.

During LTSP, it has been shown that continuously evolving populations can follow different trajectories, likely due to the complex environment and multiple fitness peaks that novel mutants can exploit (8). One method of identifying and characterizing these beneficial mutations is to observe the growth advantage in stationary phase (GASP) phenotype, which is defined as the ability of cells isolated from aging cultures to outcompete cells from younger cultures when coincubated, due to the acquisition of beneficial mutations during LTSP (9–11). Within an initially isogenic population, GASP alleles can be detected within 10 days of batch incubation (5, 7, 8). The best-characterized mutations that confer a GASP fitness advantage during LTSP have been identified in rpoS (7), encoding the stationary-phase or starvation-specific sigma factor, and lrp (10), a transcription factor shown to share many common regulated genes with RpoS (12). Analysis of *rpoS* and *lrp* GASP mutants has shown that these mutations allow E. coli to better utilize amino acids as carbon sources (7, 9). More recently, it was shown that mutations in *cytR*, *sspA*, and *tolC* fix in as few as 30 generations in populations incubated in rich medium and serially passaged after entering LTSP (3). These genes encode proteins involved in nucleoside catabolism, the stringent starvation response, and transport of toxic compounds, respectively. Complex mutations involving mobilization of insertion sequence (IS) elements have also been shown to confer the GASP phenotype (11). In addition to these well-characterized mutations, other observations suggest that many more GASP alleles exist (13).

Two recent studies examined the genetic composition of populations during LTSP: one over the course of 28 days and another over a period of 4 months (14, 15). However, what remains poorly understood in the long-term batch culture system is just how complex evolving populations can become over longer periods of time, how many genotypes can be present at any one time, and the frequency of genetic sweeps through the community. To address these questions, we extensively characterized an evolving population of E. coli, inoculated from a single clone, that was incubated in batch culture for 1,200 days without the addition of nutrients. The genomes of 1,117 clones isolated across 24 time points were individually sequenced to identify the precise mutations present in each clone during the evolution experiment. Together, these mutations allowed us to determine the underlying population structure and evolutionary paths observed within the community.

## RESULTS

### Whole-genome resequencing of clones from 24 time points across 1,200 days.

**FIG 1:**
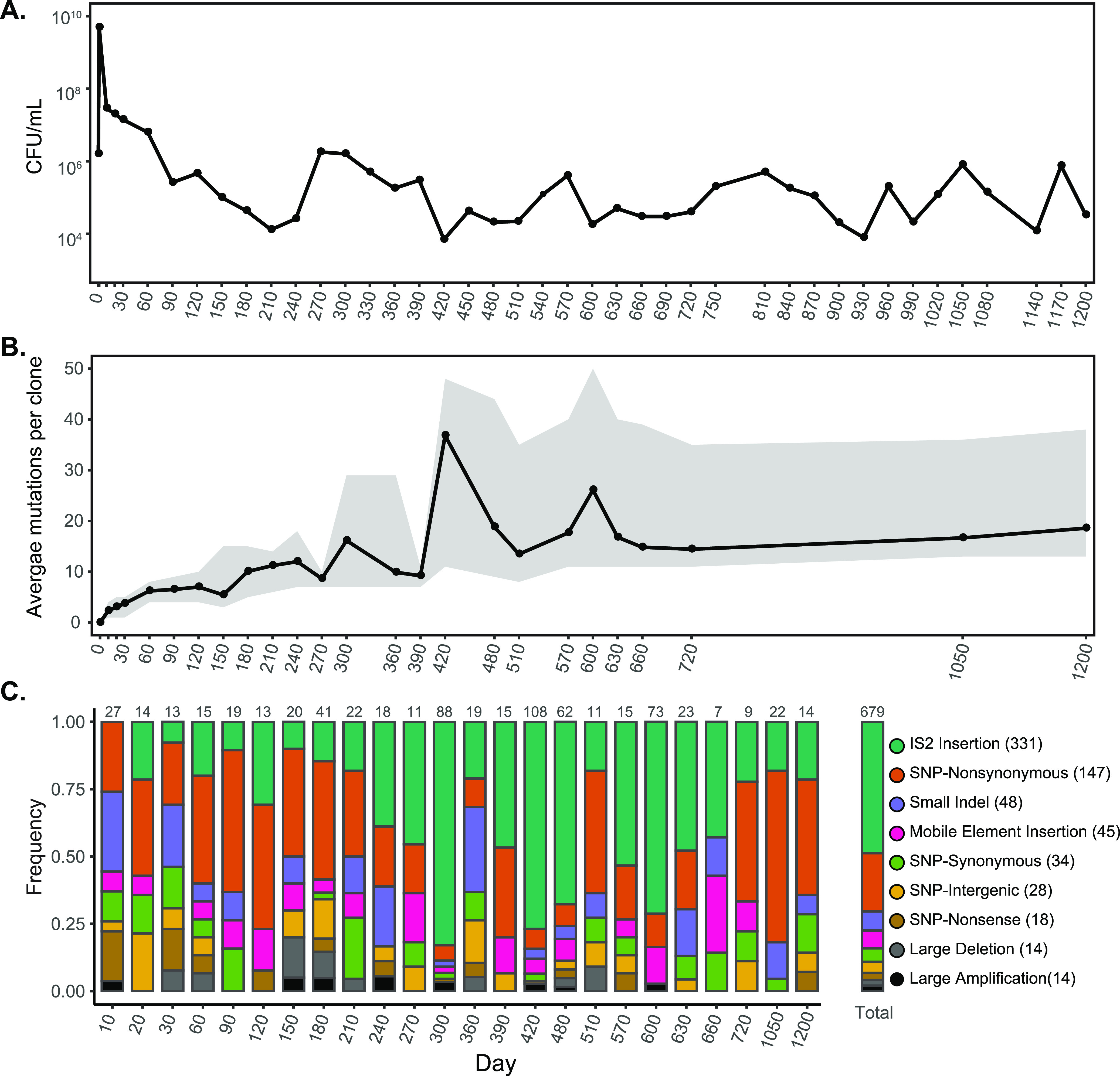
Population density, number, and frequency of mutations detected in E. coli during 1,200 days of incubation in a long-term batch-culture evolution experiment. (A) The population density is shown as CFU/ml on a semi-log plot. (B) The average number of mutations per clone for each time point where clones were sequenced is graphed with the range shown in gray. (C) The frequencies of identified IS2 insertions, nonsynonymous mutations, small indels ranging from 1 to 16 bp, mobile element insertions, synonymous mutations, intergenic SNPs, nonsense mutations, large deletions ranging from 100 bp to 64 kbp, and large amplifications are shown for the 1,117 clones that were sequenced across 24 time points. The frequency of each mutation type is also shown for all time points. The number of mutations identified is shown above each bar.

A single strain of E. coli was incubated in batch culture for 1,200 days, and 1,117 clones isolated throughout the experiment were sequenced. The batch culture was initiated by inoculating approximately 10^6^ CFU into 5 ml of LB. Ten microliters of the population was frozen on days 10, 20, and 30 and every subsequent 30 days. The population experienced the five growth phases typical of E. coli K-12 incubated in LB (5) (Fig. 1A). The population density increased more than 1,000-fold to 5 × 10^9^ CFU/ml after exponential growth on day 1. Following stationary phase and death phase, the population size remained around 5 × 10^7^ CFU/ml between days 10 and 60. After day 60, the LTSP population density ranged from 10^4^ to 10^6^ CFU/ml (Fig. 1A).

To examine the population structure of the long-term batch culture at the genotypic level, ∼48 clones from each time point were isolated on LB agar plates from the frozen samples and individually sequenced. An average of 40-fold sequence coverage was obtained per clone using Illumina NextSeq and HiSeq technology. The number of mutations per clone generally increased over time, with clones from day 1200 having acquired an average of 18 mutations (Fig. 1B). We identified 679 unique mutations across all clones, including 147 nonsynonymous single nucleotide polymorphisms (SNPs) in protein-coding genes, 48 small indels (1 to 16 bp), 376 new insertion sequence (IS) elements, 14 large deletions (100 bp to 64 kbp), and 14 duplications/amplifications (∼50 to ∼500 kbp) (Fig. 1C). Of the 376 novel IS element insertions identified, 331 are exclusively IS2 elements, and most occurred between days 270 and 600 (Fig. 1C) in only one lineage (see the discussion on IS2 below).

The spectrum of de novo SNPs favored G:C > A:T (see Fig. S1 in the supplemental material), which has been observed in other studies (14, 16, 17). The overall spectrum of mutations is significantly different compared to the mutation accumulation study performed by Lee et al. (16). However, the mutation spectrum from this work is more similar to another long-term batch culture evolution experiment, with an underrepresentation of A:T > T:A compared to Lee et al. (16), suggesting differences are due to incubation conditions (growth on plates versus liquid culture) (14).

10.1128/mBio.03337-20.2 FIG S1 Mutation spectrum distribution. The distribution of the six possible base pair changes are shown for 227 SNPs identified from the 1,117 sequenced clones (dark gray) and Lee et al. (16). (light gray). By chi-square contingency test, P = 4.95e−07. Download FIG S1, PDF file, 0.1 MB. Copyright © 2021 Ratib et al. 2021 Ratib et al. This content is distributed under the terms of the Creative Commons Attribution 4.0 International license.

### Four major lineages were observed over 1,200 days of evolution.

**FIG 2:**
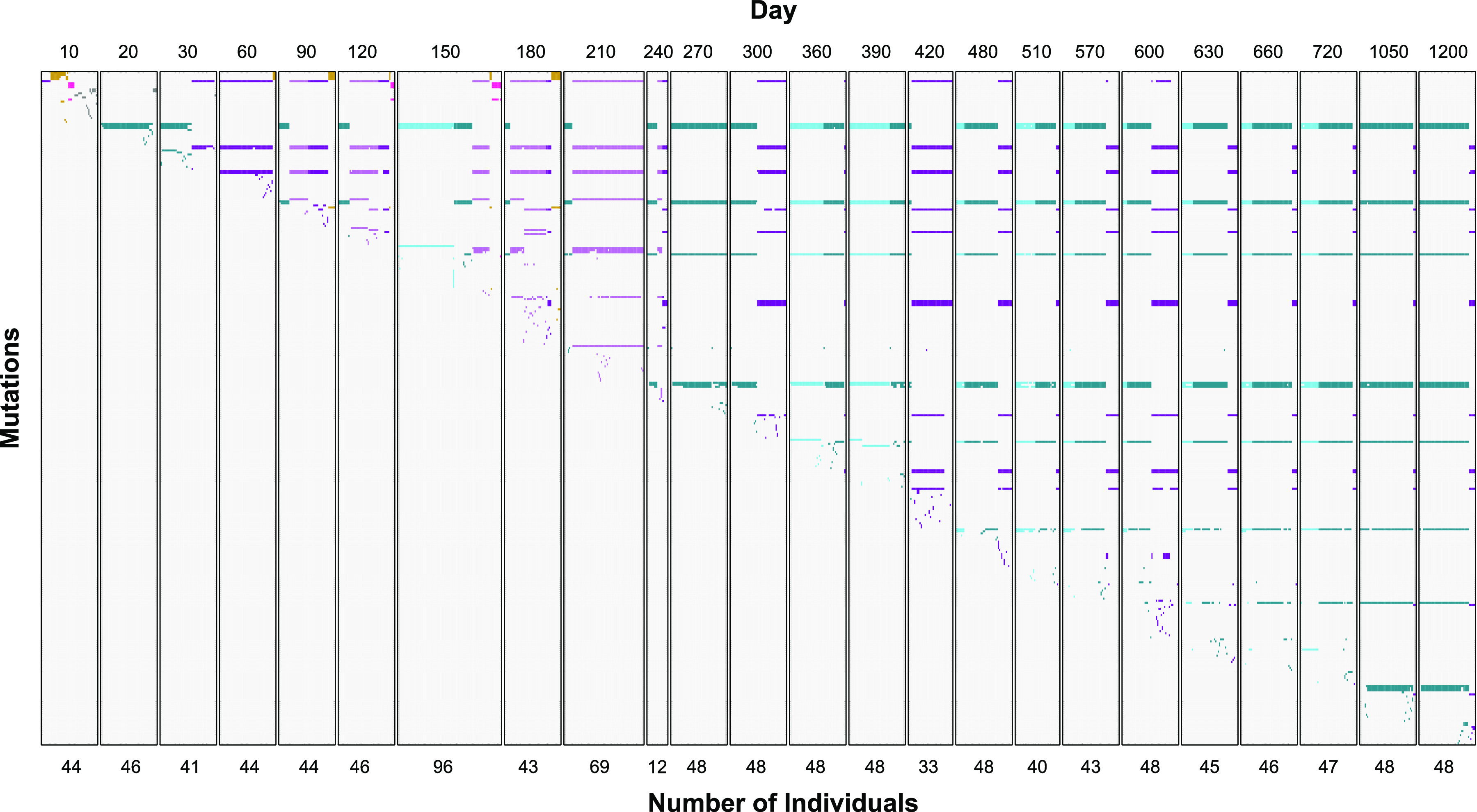
All mutations present (excluding IS2 insertions) in individual clones from days 10 to 1200. Time points are separated into panels, where columns represent individual clones and rows are specific mutations. Days are indicated at the top of each panel, and the number of clones sequenced is indicated at the bottom. Each colored box represents a mutation identified in a clone, while uncolored boxes represent wild-type alleles. Column colors indicate the lineage a clone belongs to (blue, L1; purple, L2; orange, L3; pink, L4; gray, early genotypes not detected after 30 days). All mutations identified are presented in Data Set S1.

**FIG 3:**
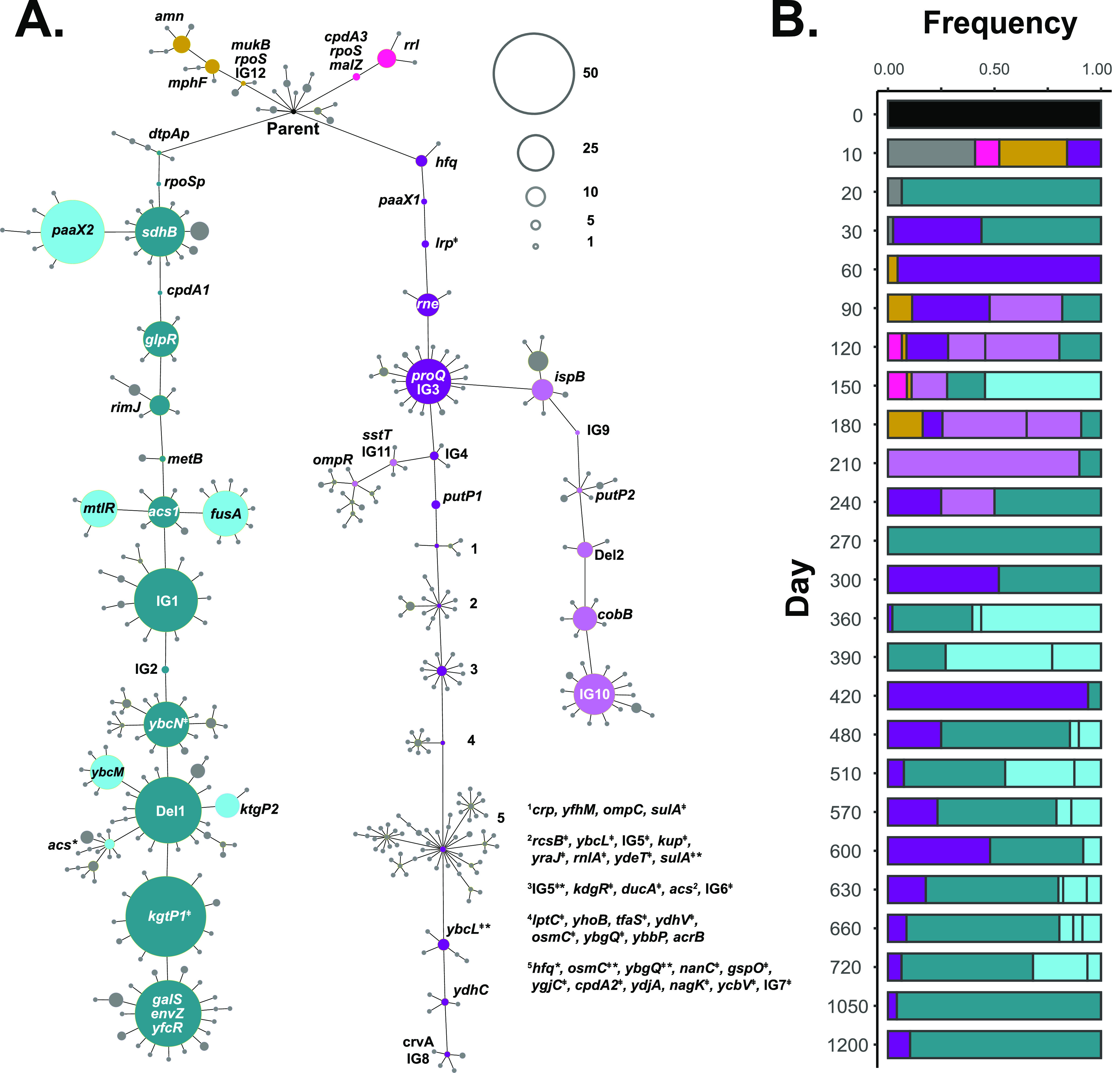
Four main lineages diverge from the parent between days 10 and 1200. (A) A minimum-spanning tree showing phylogenetic relatedness of all 1,117 clones sequenced. Each node represents a single genotype, with its size proportional to the number of clones with that particular genotype; unfilled circles in the upper right provide a reference scale for the number of clones. The parental genotype is indicated as the black node at the top. Gene names within or adjacent to nodes indicate the mutated gene(s) for that genotype. The four main lineages are indicated by color (L1, blue; L2, purple; L3, orange; L4, pink.) Sublineages that diverge are indicated in lighter shades of blue and purple. Collateral genotypes are indicated in gray. Genes mutated for genotypes 1 to 5 within L2 are listed to the right of the tree. ‡ indicates new insertion element (IS). * indicates reversion to wild-type allele. (B) Frequency of main lineage clones for each time point.

The population structure of the evolving community was highly dynamic during incubation in long-term batch culture. Four major lineages (L1 to L4) diverged from the parental genotype and were present 150 days or longer (Fig. 2 and 3). Most sequenced clones belonged to L1 (blue [61%]) and L2 (purple [33%]), which were present through day 1200, while L3 (orange [3%]) and L4 (pink [1%]) were present as a minority through days 180 and 150, respectively (Fig. 2 and Fig. 3B).

10.1128/mBio.03337-20.9 DATA SET S1 Mutations present in clones from cultures 1 to 4. Download Data Set S1, XLSX file, 0.05 MB. Copyright © 2021 Ratib et al. 2021 Ratib et al. This content is distributed under the terms of the Creative Commons Attribution 4.0 International license.

After 10 days in long-term batch culture, novel mutants arose and became the founding genotypes for three of the four lineages observed throughout the duration of the experiment (Fig. 2). Ten days later on day 20, the population structure shifts with nearly all clones (45 out of 48) belonging to L1 and sharing the same three mutations affecting the dtpA promoter, which encodes a di- and tripeptide transporter, the rpoS promoter, the stationary-phase-specific sigma factor, and the coding region of sdhB, encoding succinate dehydrogenase. After another 10 days (day 30) the population structure shifts again, with half of the population belonging to L2—the majority of these clones sharing two new mutations compared to their day 10 ancestors. By day 60, nearly the entire population belongs to L2, where two additional mutations have fixed compared to day 30. L1 is again detected on day 90 after acquiring two additional mutations, and L2 diverges into two sublineages, which include 10 or more clones, that are present until day 240 (Fig. 2; light purple). L1 also has transient sublineages that are observed on day 150, as well as days 360 through 720 (Fig. 2; light blue). The observed relative frequencies of L1 versus L2 in the population does not correlate with whether the overall population density experienced a recent increase or decrease (see Fig. S2 in the supplemental material).

10.1128/mBio.03337-20.3 FIG S2 Relative abundance of L1 and L2 in the population. The CFU/ml of the total population (black), L1 (blue), and L2 (purple) is shown on a semi-log scale. Open circles represent when lineages are undetected. Download FIG S2, PDF file, 0.1 MB. Copyright © 2021 Ratib et al. 2021 Ratib et al. This content is distributed under the terms of the Creative Commons Attribution 4.0 International license.

### Genetic complexity and selective sweeps in L1 and L2.

**FIG 4:**
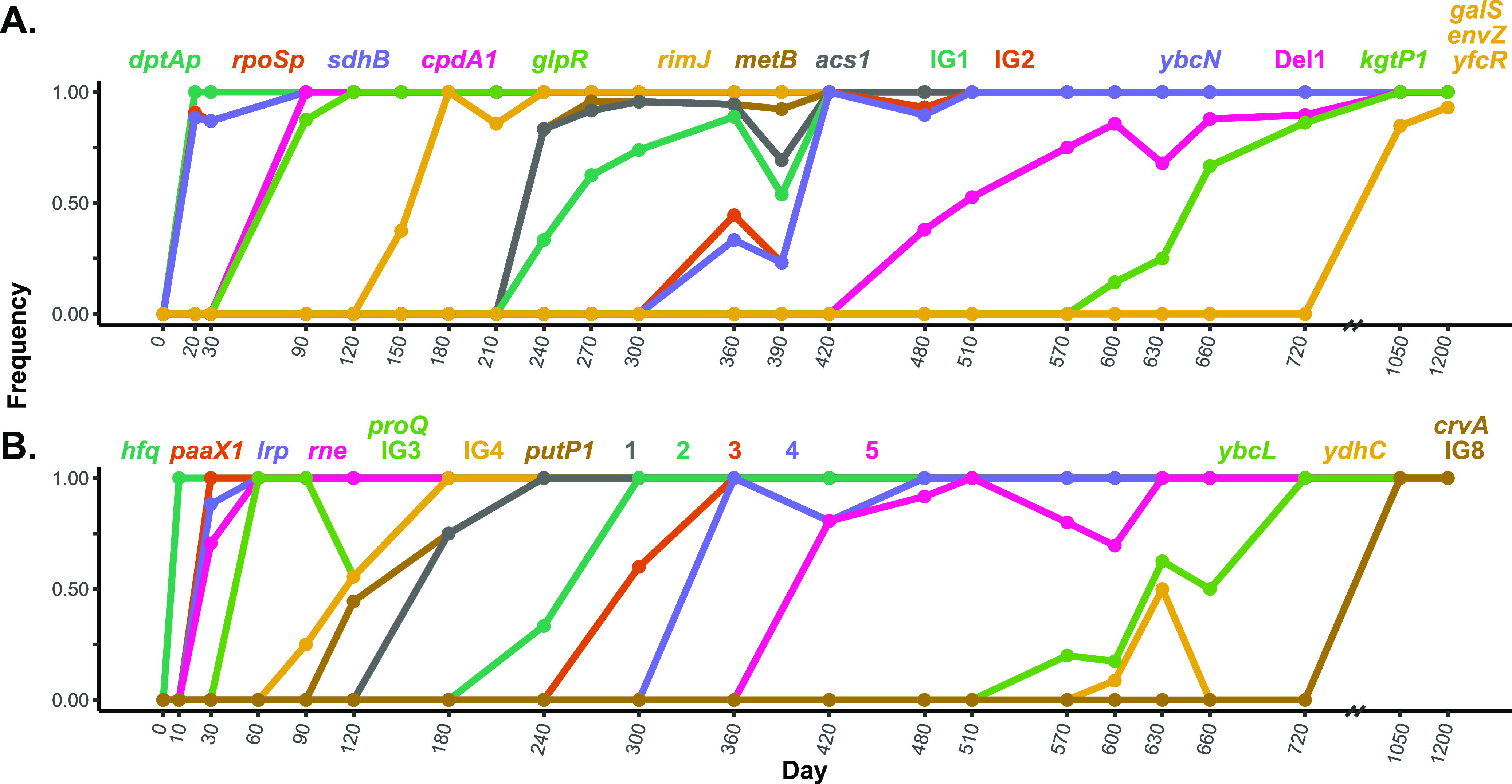
Allele frequencies of core mutations within lineages 1 and 2 during evolution in LTSP. Allele frequencies for novel mutations appearing in clones from lineage 1 (A) and lineage 2 (B) are shown. Mutated genes are indicated above the plots, with color coordinating to line color. For L2, genes in genotypes are noted as follows: 1, crp, yfhM, ompC, and sulA^‡^; 2, rcsB^‡^, ybcL^‡^, IG5^‡^, kup^‡^, yraJ^‡^, rnlA^‡^, ydeT^‡^, and sulA^‡^*; 3, IG5^‡^*, kdgR^‡^, ducA^‡^, acs, and IG6^‡^; 4, lptC^‡^, yhoB, tfaS^‡^, ydhV^‡^, osmC^‡^, ybgQ^‡^, ybbP, and acrB; and 5, hfq*, osmC^‡^*, ybgQ^‡^*, nanC^‡^, gspO^‡^, ygjC^‡^, cpdA^‡^, ydjA^‡^, nagK^‡^, ycbV^‡^, and IG7^‡^. * indicates reversion to the wild-type allele; ‡ indicates new insertion of an IS element.

Among the 1,117 clones that were sequenced, 393 unique genotypes were identified (Fig. 3A). Most genotypes belong to L1 and L2 since they dominated the population and continually acquired mutations. The evolution of L1 and L2 resulted in a series of fixation events, likely the result of positive natural selection (Fig. 4). We refer to mutations that fixed within a lineage as “core mutations” associated with that lineage and the resulting genotypes as “core genotypes.” As shown in Fig. 4, core mutations, distinguished by all clones from a given lineage having a particular mutation, regularly swept through the L1 and L2 populations. In L1 (Fig. 4A), at least 14 sweeps of core mutations occurred, roughly every 90 days, starting on day 20 with a mutation in the *dtpA* promoter. Multiple core genotypes within L1 were observed to coexist at any given time. However, ancestral genotypes were eventually outcompeted by younger genotypes. We observed the greatest genetic complexity in L1 from days 360 to 390, when at least five core genotypes coexisted, reflecting the apparent competitive advantage conferred by the sequential acquisition of mutations in *rimJ*, *metB*, *acs1*, and *ybcN* and two intergenic mutations. All six mutations ultimately become fixed by day 420.

We observed similar patterns occurring in L2, where at least 15 selective sweeps occurred. One fundamental difference between the L1 and L2 lineages is that L2 experienced substantial insertion sequence mobilization (Fig. 3A; see Fig. S3 in the supplemental material), where 21 new IS2 element insertions fixed among several genotypes. Between days 10 and 240, most fixation events involved single mutations (Fig. 4B). However, when the IS2 mobilization is first observed on day 240, as many as eight mutations fixed at the same time, most being novel IS2 elements. Any selective benefit associated with the presence of these novel IS2 elements remains to be determined.

10.1128/mBio.03337-20.4 FIG S3 Novel IS2 insertions present in individual clones from days 10 to 1200. Time points are separated into panels, where columns are clones and rows are novel IS2 insertions. Days are indicated at the top of each panel, and the number of clones sequenced is indicated at the bottom. Each colored box represents an identified IS2 in a clone, while uncolored boxes represent no IS2 insertion. Column colors indicate the lineage a clone belongs to (blue, L1; purple, L2; orange, L3; pink, L4; gray, early genotypes not detected after 30 days). All IS2 insertions identified are listed in Data Set S1. Download FIG S3, PDF file, 1.8 MB. Copyright © 2021 Ratib et al. 2021 Ratib et al. This content is distributed under the terms of the Creative Commons Attribution 4.0 International license.

Along with core genotypes, we observed many “collateral” genotypes, distinguished from core genotypes by one or more mutations that are ultimately evolutionary “dead ends.” Most of the collateral genotypes were identified only once in a single clone that diverged from the core L1 and L2 genotypes (Fig. 3A; gray genotypes). However, some of these collateral genotypes have been categorized as sublineages because they are briefly present at a higher frequency, suggesting that these genotypes were potentially, at least transiently, under positive selection (five in L1 and two in L2 [Fig. 3; light blue and purple]). Collateral genotypes could represent neutral passenger mutations, or they could have been under positive selection at least temporarily. Some collateral genotypes possess novel mutant alleles in the same genes as the core genotypes, such as two alleles in *ktgP*, encoding an α-ketoglutarate transporter in L1, supporting the idea that some mutations may have been selected (Fig. 3A).

### Types of genes where mutations fix in evolved lineages.

Many of the core mutations in the four lineages occur in genes from three broad functional groups: metabolic enzyme genes, transcriptional regulator genes, or metabolite transport genes (Table 1). Genes involved in central metabolism include sdhB, encoding a subunit of succinate dehydrogenase, acs, encoding an acetyl-coenzyme A (acetyl-CoA) synthetase, and *mhpF*, encoding an acetaldehyde dehydrogenase. Mutations in transcriptional regulators encoded by *glpR* and paaX may indirectly affect metabolism since they regulate genes involved in the catabolism of glycerol-3-phosphate and phenylacetate, which can be derived from phenylalanine degradation (18–21). Genes encoding the global transcriptional regulators RpoS, Hfq, Lrp, and CRP (cAMP receptor protein) are also mutated, potentially affecting expression of hundreds of genes (22–24).

**TABLE 1:** Lineage 1 and 2 fixed mutations

Gene^*a*^	Promoter	Mutation^*b*^	Ref codon^*c*^	New codon	Function
Lineage 1					
	*dtpA*	A→T			Dipeptide and tripeptide permease A
*nlpD*	*rpoS*	S210S	TCT	TCC	Stress-related alternative sigma factor
*sdhB*		M107I	ATG	ATA	Succinate dehydrogenase, FeS subunit
*cpdA1*		S222L	TCG	TTG	3′,5′-cAMP phosphodiesterase
*glpR*		N91fs			Repressor of the glycerol-3-phosphate regulon
*rimJ*		A61fs			Ribosomal-protein-S5-alanine N-acetyltransferase
*metB*		P146S	CCA	TCA	Catalyzes a γ-replacement reaction that produces l-cystathionine and succinate from the substrates O-succinylhomoserine and l-cysteine
*acs1*^*d*^		GTTATCGAC_1→2_			Acetyl-CoA synthetase
IG1 *mak*/*araJ*	*araJ*	G→A			Manno(fructo)kinase/l-arabinose-inducible putative transporter, MFS family
IG2 *mtlA*/*yibI*		1-bp insertion			Mannitol-specific PTS enzyme: IIA, IIB and IIC components/DUF3302 family inner membrane protein
*ybcN*		IS2 insertion			DLP12 prophage; DNA base-flipping protein
IS2 deletion		1,336-bp deletion			Downstream of *priA*, upstream of ftsN
*kgtP1*		IS3 insertion			α-Ketoglutarate transporter
*galS*		R5C	CGT	TGT	Transcription factor that represses transcription of the operons involved in transport and catabolism of d-galactose
*envZ*		R214C	CGT	TGT	Sensory histidine kinase in two-component regulatory system with OmpR
*yfcR*		V54I	GTA	ATA	Putative fimbrial-like adhesin protein

Lineage 2					
*hfq*		I44S	ATC	AGC	RNA-binding protein that stimulates RNA-RNA pairing and affects many cellular processes
*paaX1*		R74C	CGC	TGC	Transcriptional repressor that participates in controlling transcriptional regulation of genes involved in the catabolism of phenylacetic acid
*lrp*		IS2 insertion			Transcriptional regulator involved in amino acid biosynthesis and catabolism, nutrient transport, pilus synthesis, and other cellular functions
*rne*		G174D	GGC	GAC	RNA-binding protein, RNA degradosome binding protein, endoribonuclease
*proQ*		R80C	CGT	TGT	RNA chaperone, putative ProP translation regulator
*IG3 insH1*/*lnt*	*insH1*	1-bp deletion			IS5 transposase and transactivator/apolipoprotein N-acyltransferase
IG4 *clpX*/*lon*	*lon*	IS186 insertion			ATPase and specificity subunit of ClpX-ClpP ATP-dependent serine protease/ATP-dependent protease responsible for degradation of misfolded proteins
*putP1*		A337T	GCG	ACG	Sodium/proline symporter responsible for the uptake of proline
*crp*		E55K	GAA	AAA	cAMP-activated global transcription factor, mediator of catabolite repression
*yfhM*		G685D	GGC	GAC	α2-Macroglobulin, which serves to protect the cell from host peptidases
*ompC*		A282S	GCT	TCT	Outer membrane porin protein C
*rcsB*		IS2 insertion			Response regulator involved in the regulation of the synthesis of colanic acid capsule, cell division, periplasmic proteins, motility, biofilm formation, and a small RNA
*kup*		IS2 insertion			Potassium ion uptake under hyper-osmotic stress at low pH
*yraJ*		IS2 insertion			Putative chaperone-usher fimbrial protein
*rnlA*		IS2 insertion			Toxin of a toxin-antitoxin system
*ydeT*		IS2 insertion			Fimbrial usher domain-containing protein
*kdgR*		IS2 insertion			Transcriptional repressor of genes involved in transport and catabolism of 2-keto-3-deoxy gluconate (KDG)
*dcuA*		IS2 insertion			C_4_-dicarboxylate antiporter required for the uptake of l-aspartate as a nitrogen source under aerobic conditions
*acs2*		M1V	ATG	GTG	Acetyl-CoA synthetase
IG6 *arpB*/*yniD*	*yniD*	IS2 insertion			Pseudogene putative ankyrin repeat protein B, N-terminal fragment/uncharacterized protein; contains a predicted transmembrane segment
*lptC*		IS2 insertion			LPS export protein, periplasmic membrane-anchored LPS-binding protein
*yhbO*		A113D	GCC	GAC	Stress-resistance protein
*tfaS*		IS2 insertion			CPS-53 (KpLE1) prophage; tail fiber assembly protein fragment
*ydhV*		IS2 insertion			Putative oxidoreductase subunit
*ybbP*		D54E	GAT	GAA	Putative ABC transporter permease
*acrB*		IS2 insertion			Multidrug efflux system protein
*nanC*		IS2 insertion			N-Acetylnuraminic acid outer membrane channel protein
*gspO*		IS2 insertion			Bifunctional prepilin leader peptidase/methylase
*yqjC*		IS2 insertion			DUF1090 family putative periplasmic protein
*cpdA2*		IS2 insertion			3′,5′-cAMP phosphodiesterase
*ydjA*		G129G	GGC	GGT	Putative oxidoreductase
*nagK*		IS2 insertion			*N*-Acetyl-d-glucosamine kinase
*ycbV*		IS2 insertion			Putative fimbrial-like adhesin protein
IG7 *yjjY*/*yjtD*	*yjtD*	IS2 insertion			Uncharacterized protein/putative methyltransferase
*ydhC*		P227L	CCG	CTG	Putative arabinose efflux transporter
*cvrA*		F311fs			Putative cation/proton antiporter
IG8 *phoE*/*proB*	*phoE*	IS2 insertion			Outer membrane phosphoporin protein E/gamma-glutamate kinase

Lineage 3					
*cpdA3*		3-bp deletion			3′,5′-cAMP phosphodiesterase
*rpoS*		1-bp deletion			RNA polymerase, sigma S (sigma 38) factor
*malZ*		I128T	ATC	ACC	Maltodextrin glucosidase
*rrlH*		1-bp insertion			23S ribosomal RNA of rrn operon

Lineage 4					
IG12 *psuK*/*fruA*	*psuK*	A→C			Pseudouridine kinase/fused fructose-specific PTS enzymes: IIB component/IIC components
*mukB*		R1465R	CGA	CGG	Chromosome condensin MukBEF, ATPase and DNA-binding subunit
*rpoS*		L116*	TTG	TAG	RNA polymerase, sigma S (sigma 38) factor
*mhpF*		T55T	ACC	ACT	Acetaldehyde-CoA dehydrogenase II, NAD-binding
*amn*		1-bp insertion			AMP nucleosidase

a Gene names for mutations affecting coding regions are listed, and the two genes adjacent to the intergenic (IG) mutations listed are separated by “/.”

b Amino acid changes are listed for synonymous and nonsynonymous mutations. Nucleotide changes are listed for intergenic mutations. The number of nucleotides inserted or deleted is listed for indels. IS insertions are listed with the type of IS.

c “Ref codon” refers to the reference codon.

d The acs1 mutation is the result of a 9-bp duplication. The repeated nucleotide sequence is listed under the column heading “Mutation.”

Core mutations in genes encoding membrane proteins involved in the transport of small peptides and amino acids include *putP*, encoding a sodium/proline symporter, ybbP, encoding a predicted component of an ABC superfamily metabolite uptake transporter, and sstT, encoding a sodium ion-coupled serine/threonine symporter. Both envZ and ompR, a two-component system controlling expression of the outer membrane porin genes *ompC* and *ompF*, are mutated in L1 and L2 (Table 1) (25). Mutations in *envZ*, *ompC*, and *ompR* all fix within L1, L2, and a sublineage of L2, respectively (Fig. 3A). Normally, expression of ompF decreases as cells transition from exponential to stationary phase in an rpoS-dependent manner (26).

We also identified core mutations that potentially affect promoters and downstream gene expression. The first mutation appearing in L1 is an intergenic mutation upstream of dtpA, encoding a di- and tripeptide transporter (27) (Table 1). The A-to-T mutation is adjacent to the −10 sequence, and quantitative reverse transcription-PCR (qRT-PCR) shows that dptA mRNA levels in clones with this mutation are increased ∼23-fold (see Fig. S4 and Text S1 in the supplemental material). The second mutation to fix within L1 alters the promoter of rpoS (Table 1). The T-to-C change identified within the −10 sequence of rpoS results in an 8-fold decrease in expression of rpoS, as measured by qRT-PCR (Fig. S4 and Text S1). Both of these transcriptional changes might be associated with an increased capacity to import peptides and metabolize amino acids in these peptides as sources of carbon and energy. The first GASP mutant identified during long-term batch culture was an rpoS allele with a 46-bp duplication, resulting in decreased RpoS activity that allowed cells to utilize amino acids as a sole carbon source better than the parent (9).

10.1128/mBio.03337-20.1 TEXT S1 Methods for qRT-PCR to measure gene expression, glycogen and catalase assays to measure RpoS activity, and subsampling to determine the number of clones to sequence. Download Text S1, PDF file, 0.1 MB. Copyright © 2021 Ratib et al. 2021 Ratib et al. This content is distributed under the terms of the Creative Commons Attribution 4.0 International license.

10.1128/mBio.03337-20.5 FIG S4 Relative gene expression for clones with mutations in promoter regions of dtpA and rpoS. qRT-PCR was used to measure the relative transcript abundance of dtpA and rpoS compared to the wild type. Expression is shown on a log_2_ scale (Methods are presented in Text S1.). Download FIG S4, PDF file, 0.02 MB. Copyright © 2021 Ratib et al. 2021 Ratib et al. This content is distributed under the terms of the Creative Commons Attribution 4.0 International license.

### IS2 element mobilization in L2.

New IS element insertions, which can result in disrupted gene function if located within a coding or regulatory region, were also identified in evolved clones (28). A substantial increase in the number of IS2 transposition events was first detected on day 300, when the frequency of L2 clones increased to nearly half of the population (Fig. S3). The IS2 insertions occur nearly exclusively within L2, suggesting the mobilization is likely due to either a physiologic or genetic response. IS2 has been shown to transpose via a two-step “copy-and-paste” mechanism (29, 30), with CRP negatively regulating this process by binding the promoter of the IS2 element transposase gene (31). Prior to the expansion of IS2 within L2, a single mutation in crp had fixed, which may be the causal factor in the IS2 mobilization (Fig. 4B; genotype 1). By day 1200, 21 novel IS2 elements fixed within the genome of L2 cells, 18 of which inserted in protein coding regions (Table 1).

Overall, of the 331 independent IS2 insertion events observed, 19% (63/331) inserted into noncoding regions and 81% (268/331) inserted into coding regions. Since 90% of the parental genome is coding, significantly more IS*2*s inserted into noncoding regions than expected (chi-square test, P ul> 0.0001), suggesting that some insertions in coding regions are deleterious and therefore selected against. The number of novel IS2 insertions in L2 decreases after day 600 (Fig. S3), corresponding with the fixation of an IS2 insertion into cpdA (Fig. 4B; genotype 5), encoding a cAMP-phosphodiesterase. This mutation potentially affects the level of cAMP in the cell, which modulates CRP activity, thus downregulating IS2 transposase activity (32). This hypothesis could be tested by engineering strains with these mutations and comparing the frequency of IS2 element insertion events.

### Large genomic amplifications and deletions.

A total of 14 distinct large-scale genomic amplification events affecting >1% of the chromosome and 14 large deletions ranging from 0.1 to 64 kbp were detected over the course of the experiment (see Fig. S5 in the supplemental material). All large amplifications and 13 of the 14 large deletions were detected at only a single time point. The sole large deletion to fix within a major lineage was the result of a parental IS2 excision, which fixed within L1 (Table 1). Since these large amplifications and deletions were transient, we did not observe any significant change in overall genome size over time, unlike those observed in some serial passage evolution experiments (33). We hypothesize that the complex mixture of potential energy sources in the LTSP environment makes large-scale genomic losses maladaptive over longer time periods. Cells surviving during LTSP are more likely to utilize many different energy sources, requiring maintenance of genes associated with their use, whereas gene loss in a serial passage regimen with a single energy source is less likely to be maladaptive if those genes are for utilization of an unavailable energy source.

10.1128/mBio.03337-20.6 FIG S5 Examples of 14 unique amplifications identified from each time point. Coverage across the genome of clones is shown relative to the average coverage for 14 clones with the unique amplifications identified. Increases in read depth between 2- and 5-fold were observed for various regions across the genome. Download FIG S5, PDF file, 0.1 MB. Copyright © 2021 Ratib et al. 2021 Ratib et al. This content is distributed under the terms of the Creative Commons Attribution 4.0 International license.

### Identification of parallel evolution.

**FIG 5:**
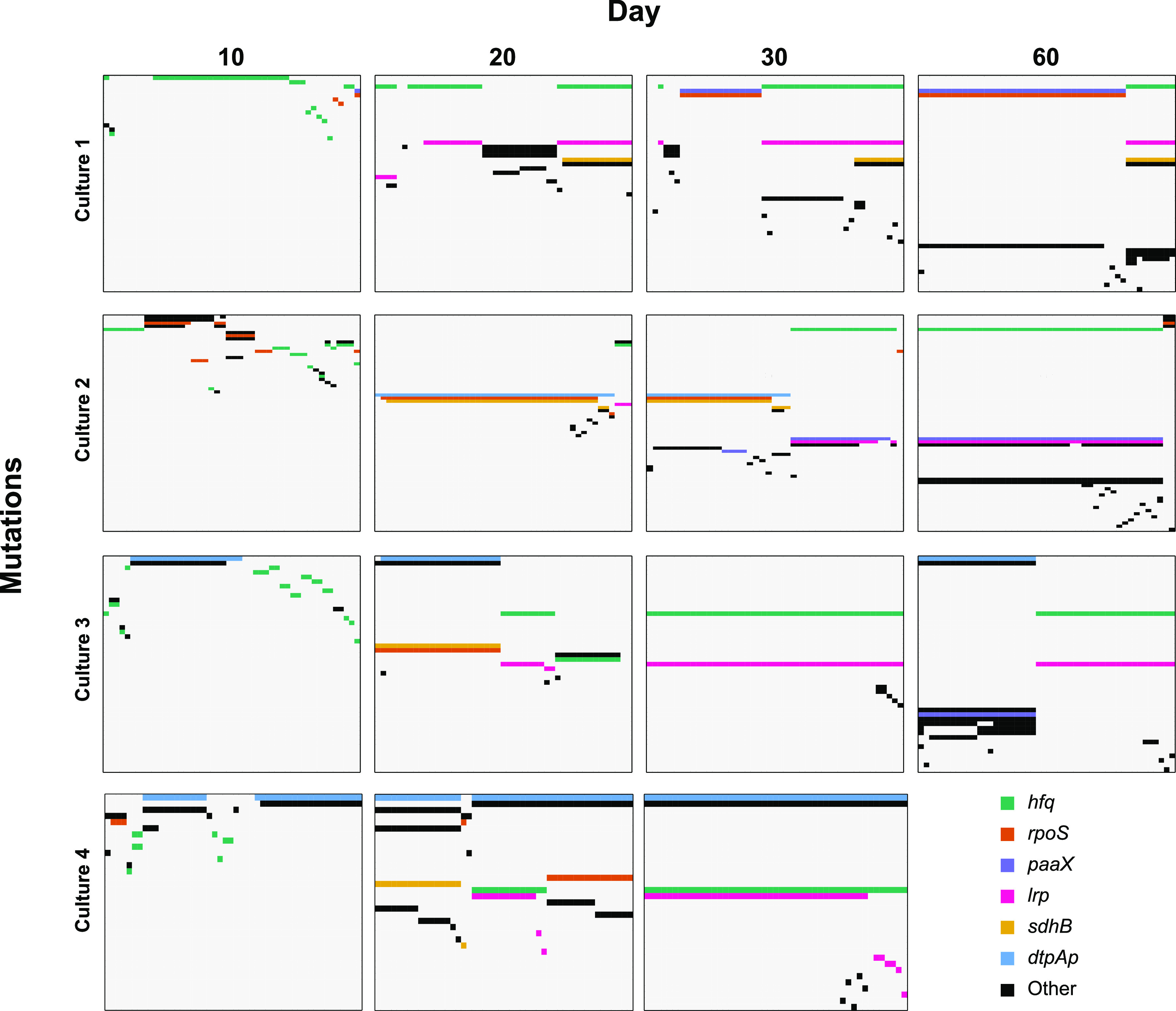
All mutations present in individual clones for up to 60 days in four parallel cultures. Cultures are separated by panel rows, and time points are separated by panel columns. Within each panel, columns represent individual clones and rows are specific mutations. Each colored box represents a mutation identified in a clone, while uncolored boxes represent wild-type alleles. Row colors indicate the gene affected by a mutation, with the legend present in the bottom right.

To determine the reproducibility of the population dynamics and mutations identified from the population described thus far (culture 2), we sequenced clones from three additional parallel populations (cultures 1, 3, and 4) up to day 60. On days 10, 20, and 30, we sequenced 48 clones from the three additional parallel populations, and on day 60, we sequenced clones from cultures 1 and 3. We observe divergence of subpopulations that change in relative frequency and acquire mutations over time in all four experimental cultures by day 20 (Fig. 5). Over the first 60 days, mutations in hfq, rpoS, paaX, lrp, sdhB, and dtpA fix within subpopulations in at least three of the cultures, suggesting these mutations were under positive selection (Fig. 5 and Table 2). Mutant alleles of hfq and lrp are present within the same subpopulations in the four parallel cultures, with hfq mutations acquired first followed by mutations in lrp, suggesting a potential benefit of having these mutations together or a potential epistatic effect (Fig. 5).

**TABLE 2:** Genes and intergenic regions mutated in two or more cultures

Gene	No. of mutant alleles for population:				No. of alleles^*a*^		Function
	1	2	3	4	Total	Unique	
*hfq*	9	8	15	6	38	31	RNA binding protein that regulates mRNA stability and exhibits RNA chaperone activity
*rpoS*	5	5	1	2	13	11	Alternative sigma factor regulating genes involved in the general stress response
*lrp*	2	3	2	5	12	9	Regulates genes involved in amino acid biosynthesis and catabolism, nutrient transport, and pili synthesis
*sdhB*	1	2	1	2	6	5	Subunit of succinate dehydrogenase
*paaX*	2	3	1		6	6	Transcriptional repressor of genes involved in catabolism of phenylacetic acid
*dtpAp*		1	1	1	3	1	Di- and tripeptide transporter
*psuKp*	1	1	1		3	1	Pseudouridine kinase
*proQ*	1	6	1		8	8	Small RNA chaperone that post-transcriptionally regulates levels of ProP, involved in proline utilization
*glpR*	5	2			7	7	Repressor of the glycerol-3-phosphate regulon
*cpdA*	1	4			5	4	cAMP phosphodiesterase
*putA*		1		2	3	3	Proline degradation pathway
*rcsB*		3	1		4	4	Positive regulator of colanic acid biosynthesis and also an activator of *ftsZ* expression

a Total alleles are the total number of mutant alleles identified within a gene among the four populations. Unique alleles are the number of distinct mutant alleles identified within a gene among any of the four populations.

We also detected instances where the same mutation is present in multiple cultures (Table 2). For example, the same SNP within the dtpA promoter shown to result in increased dtpA expression (Fig. S4) is present in cultures 2, 3, and 4 (Table 2; see Data Set S1 in the supplemental material). There is also extensive polymorphism of the gene hfq in all four cultures, with a total of 38 different mutant alleles identified, some of which appear in multiple cultures (Fig. 5 and Table 2; Data Set S1). It is possible that instances of shared mutations could have been present in the inoculum since cultures 1 and 2 were inoculated with ZK1142 and cultures 3 and 4 were inoculated with ZK1143 (Table S1). However, there are cases of shared mutations in parallel cultures with different starting inoculums, indicating the same mutation arose multiple times independently and supporting that these alleles were strongly selected (Table 2).

10.1128/mBio.03337-20.8 TABLE S1 Isolates used for growth and competition experiments. Download Table S1, DOCX file, 0.1 MB. Copyright © 2021 Ratib et al. 2021 Ratib et al. This content is distributed under the terms of the Creative Commons Attribution 4.0 International license.

Thirteen of the mutant hfq alleles encode a nonsense mutation, while nine encode a frameshift mutation, suggesting that deactivation of Hfq was under strong positive selection (Data Set S1). Hfq is a highly conserved small RNA chaperone, functioning as a homohexamer, which regulates translation of many mRNAs, including positive regulation of rpoS translation (23, 34, 35). Thirteen mutant rpoS alleles were also detected among the four parallel cultures, four resulting in nonsense mutations, one resulting in a frameshift mutation, and three changing the isoleucine at amino acid position 128 to either asparagine or serine (Data Set S1). The hfq and rpoS alleles may all be providing an advantage by downregulating the RpoS regulon, similar to the first GASP mutation identified in LTSP batch culture (7). Assays for the production of glycogen or degradation of hydrogen peroxide are frequently used to test for RpoS activity since these processes are directly regulated by RpoS (36, 37). Clones isolated from day 10 harboring hfq and rpoS mutant alleles showed decreased RpoS activity (data not shown) when assaying the products of these stationary-phase-specific genes, supporting a model where decreased RpoS activity is under strong selection by day 10.

### Evidence of selection within culture 2.

Within culture 2, we sequenced more clones and examined more time points compared to the three parallel cultures and identified multiple alleles of several genes among the 679 mutations, providing evidence that these alleles were under positive selection (Table 3). One of the core mutations within L2 affects *putP*, coding for a proline transporter. A second putP mutation is present within an L2 collateral genotype (Fig. 4). Similarly, one of the core mutations in L1 affects *kgtP*, an α-ketoglutarate transporter gene. A second *kgtP* mutation is present within a collateral L1 genotype. However, only one mutant allele of *putP* and *kgtP* ultimately fixes within either L1 or L2, highlighting potential examples of clonal interference. Which allele ultimately fixed was likely determined by the subsequent mutations that were acquired. We also identified five alleles of *proQ*, a small RNA chaperone that affects mRNA levels of *proP*, an osmoprotectant/proton symporter capable of transporting proline and glycine betaine (38, 39). Furthermore, multiple alleles of genes linked to the tricarboxylic acid (TCA) cycle, such as *paaX*, *sdhB*, *fadB*, and *acs*, were detected, some of which fix in L1 and L2.

**TABLE 3:** Genes and intergenic regions mutated 3 or more times

Gene	No. of genes/intergenic regions^*a*^							Function
	Total	S	N	NS	Indel	IS (IS2)	Del	
*yafD*	13			1		12 (12)		Endo/exonuclease/phosphatase family protein
*dcuA*	9					9 (9)		C_4_-dicarboxylate antiporter
*hfq*	8		3	4	1			HF-I, host factor for RNA phage Q beta replication, global small RNA chaperone
*htrE*	6					6 (6)		Putative outer membrane usher protein
*proQ*	6		2	2	2			RNA chaperone, putative ProP translation regulator
*rhsJ*	5					5 (5)		Putative RHS domain-containing protein
*rpoS*	5		3	1	1			RNA polymerase, sigma S (sigma 38) factor
*cpdA*	4		1		1	2 (2)		3′,5′-cAMP phosphodiesterase
*dtpB/uspA*	4					4 (4)		Dipeptide and tripeptide permease B/universal stress global response regulator
*mqsA*	4					4 (3)		Antitoxin for MqsR toxin, transcriptional repressor
*nupX*	4	1	3					Nucleoside permease
*sulA*	4	1	1	1		1 (1)		SOS cell division inhibitor
*yafC/yafD*	4					4 (4)		LysR family putative transcriptional regulator/endo/exonuclease/phosphatase family protein
*yaiT*	4					4 (4)		Putative autotransporter
*ycgB/dadA*	4					4 (4)		SpoVR family stationary phase protein/d-amino acid dehydrogenase
*yneO*	4		1			3 (3)		Putative autotransporter-encoding sequence
*acs*	3	1		1	1			Acetyl-CoA synthetase
*ispB*	3		3					Octaprenyl diphosphate synthase
*lrp*	3					3 (3)		Leucine-responsive global transcriptional regulator
*paaX*	3		2		1			Transcriptional repressor of phenylacetic acid degradation paa operon, phenylacetyl-CoA inducer
*putP*	3		2		1			Proline:sodium symporter
*rcsB*	3		1			2 (2)		Response regulator in two-component regulatory system with RcsC and YojN
*smf*	3					3 (3)		DNA recombination-mediator A family protein
*ydbA*	3					2 (2)	1	Putative outer membrane protein
*yfjH*	3					3 (3)		CP4-57 prophage, uncharacterized protein
*yhcF*	3					3 (3)		Putative transcriptional regulator

a S, synonymous; N, nonsynonymous; NS, nonsense; Del, deletion.

In addition to parallelism, a way to quantitatively measure whether mutations are under positive selection is a *K*_a_/*K*_s_ analysis, which calculates the ratio of the number of nonsynonymous base pair substitutions per nonsynonymous site (*K*_a_) and synonymous substitutions per synonymous site (*K*_s_), with respect to amino encoding codons. A ratio of greater than 1 indicates positive selection, while a ratio of less than 1 indicates purifying selection. An analysis of codons affected by SNPs using the seqinR R package resulted in a *K*_a_/*K*_s_ value of 1.84, further supporting the hypothesis that mutations are under selection (40).

Within a single gene, numerous mutant alleles associated with insertions of IS2 elements were detected, providing examples of genes whose altered activity may be beneficial during periods of starvation (Table 3). *yafD*, encoding an endo/exonuclease/phosphatase family protein, and dcuA, encoding a C_4_-dicarboxylate transporter, are the two genes with the most mutant alleles identified (Table 3). Thirteen different mutant alleles of *yafD* were detected, 12 of which resulted from independent IS2 insertion events at different locations, as well as one nonsense mutation. Four unique IS2 insertions upstream of yafD were also identified, potentially affecting the gene’s expression. Nine mutant alleles of dcuA, all the result of different IS2 insertions, were also identified. Given that we detected 331 unique IS2 insertion events over the course of the experiment, we would expect ∼298 insertions into coding regions since 90% of the genome is coding. This would result in a roughly 7% chance for any the 4,295 genes to be affected by an IS2 insertion. However, the fact that 21 IS2 insertion mutations were found within just two genes, *yafD* (801 bp) and *dcuA* (1,302 bp), is significantly more than expected in a genome of 4,541,111 bp (Fisher’s exact test, P = 6.9e−07). The number of IS2 insertions in these genes suggests that either disrupting their function was under very strong positive selection, or these regions of the genome are IS2 insertion hot spots. Future experiments could identify any fitness advantage associated with disrupting these genes by competing a knockout mutant against the parent during long-term batch culture incubation.

### Clones from L1 and L2 are physiologically distinct.

**FIG 6:**
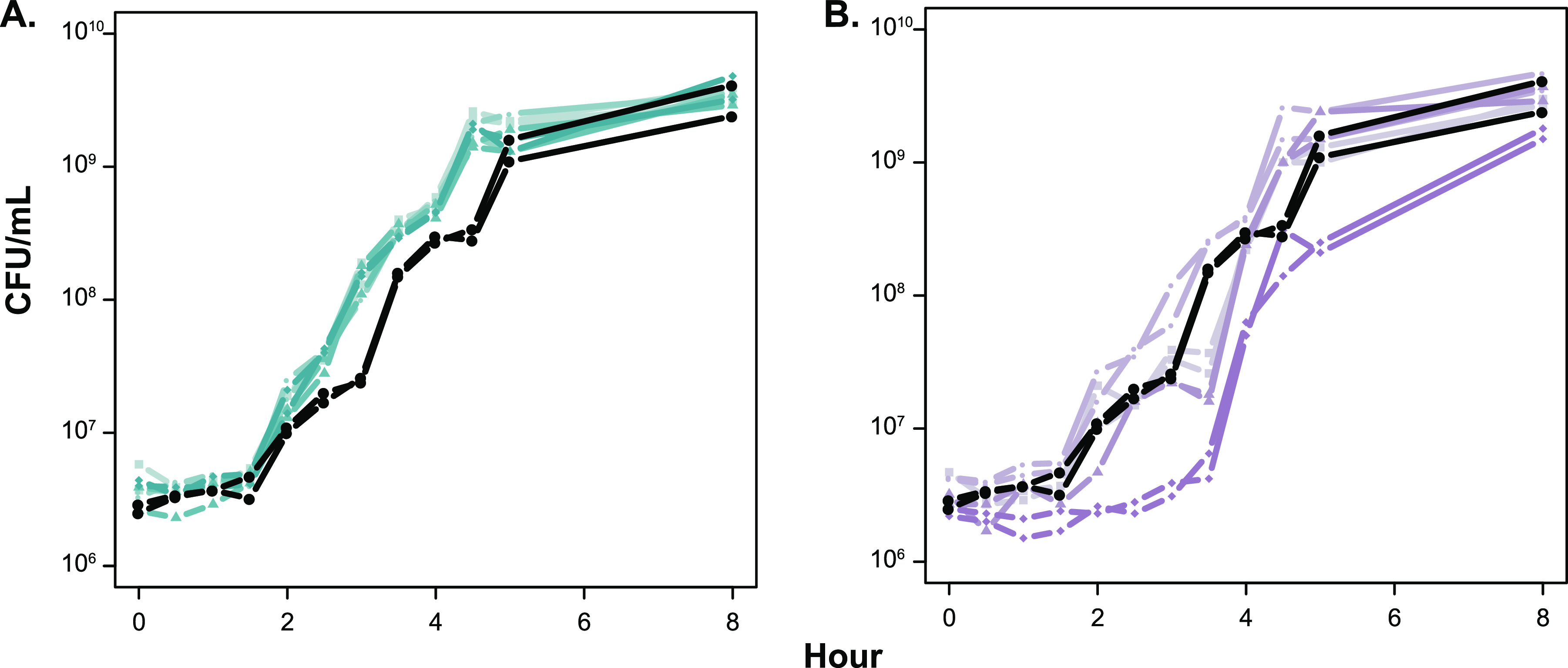
Outgrowth of isolates from L1 and L2 in LB. After inoculation into LB, titers of cultures were determined to determine the CFU/ml over a period of 8 h. Each strain is shown in duplicate, with the wild type (ZK1142) shown in black, isolates from L1 shown in blue (A), and isolates from L2 shown in purple (B). Isolates from each lineage are distinguished by different symbols: L1.1 and L2.1 by ■, L1.2 and L2.2 by ●, L1.3 and L2.3 by ▴, and L1.4 and L2.4 by ◆. Genotypes are given in Table S1.

**FIG 7:**
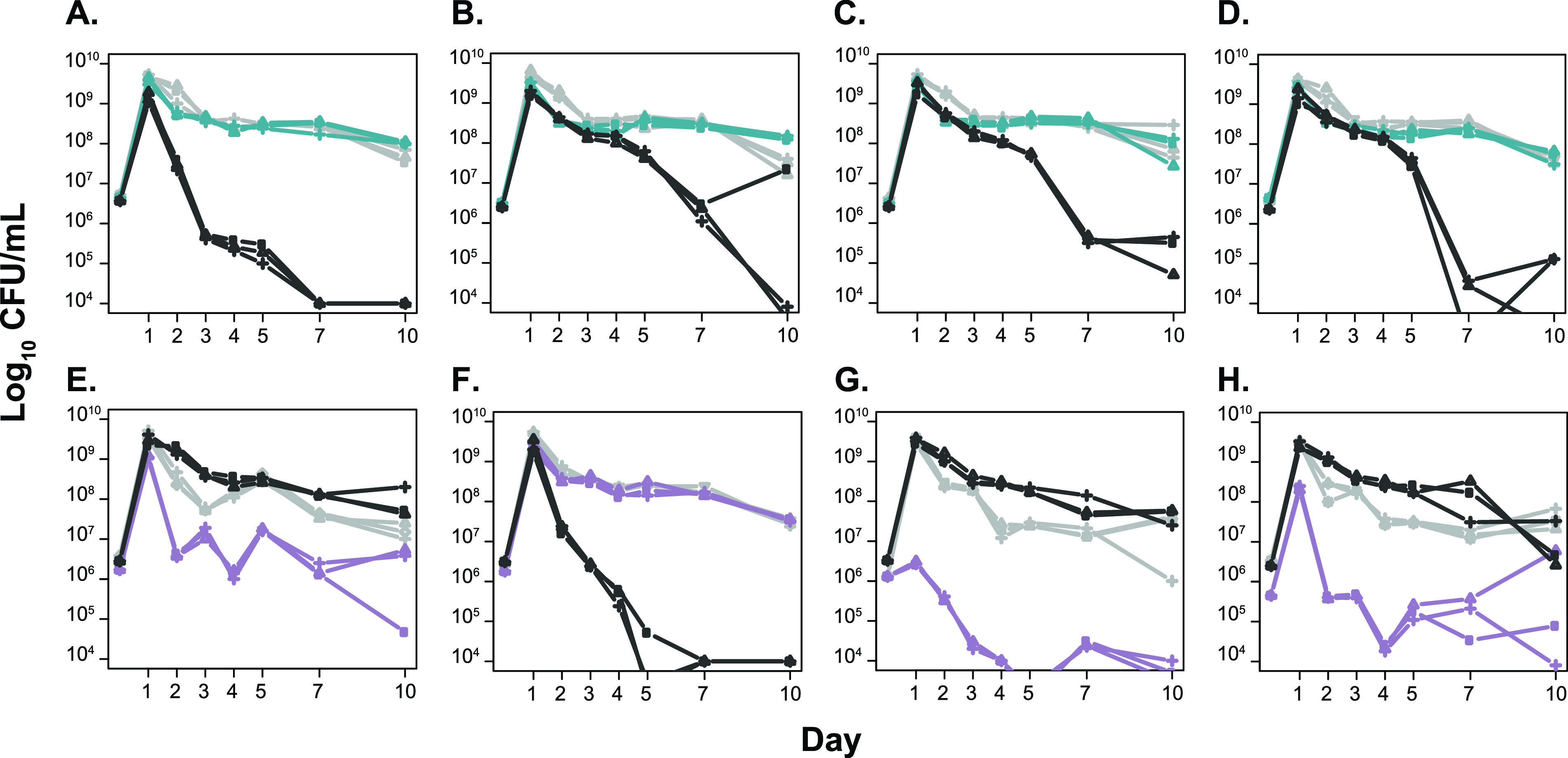
Monoculture and competition phenotypes during long-term batch culture for L1 and L2 isolates. Isolates L1.1 (A), L1.2 (B), L1.3 (C), L1.4 (D), L2.1 (E), L2.2 (F), L2.3 (G), and L2.4 (H) are shown in monoculture (gray lines for each panel) and in competition (blue or purple lines for each panel) against wild-type cells (black lines for each panel). Each is shown in triplicate. Isolate genotypes are shown in Table S1.

The coexistence of two lineages for 1,200 days suggests that these lineages diverged and evolved to occupy different niches or possibly evolved syntrophic interactions. The dynamic environment during LTSP makes it impossible to recreate the environment and selective pressures these lineages experienced during long-term incubation. Therefore, comparison of the rates of growth and survival of genomically characterized clones from L1 and L2 in the same medium environment was used to determine any phenotypic differences, which would suggest they are physiologically distinct. When grown in fresh LB, all clones from L1 have a faster doubling time than the parental strain during exponential-phase (doubling time of ∼20 min versus ∼25 min for the parent), while clones from L2 exhibit more variability in their outgrowth, with one clone having a 3-h lag phase (Fig. 6). These differences in growth support that these two lineages are physiologically distinct and possibly occupy different niches in the LTSP environment. When clones from L1 and L2 are competed against the parent in fresh medium, all L1 clones are able to outcompete the parent, while L2 clones tend to show lower relative fitness (Fig. 7), further supporting that L1 and L2 are physiologically distinct from one another.

## DISCUSSION

Our findings provide the most in-depth analysis of an E. coli population evolving during long-term batch culture to date. We identified two main subpopulations that coexisted while continuously acquiring new mutations throughout the 1,200-day incubation. Two recent studies that used whole-genome sequencing to characterize E. coli populations during long-term batch culture incubation identified mutations in subunits of RNA polymerase as opposed to mutations affecting rpoS or hfq after 10 days. These differences in mutant alleles present on day 10 are likely attributed to differences in experimental design (14, 15). These studies both incubated cell populations in relatively large volumes (200 or 400 ml compared to 5 ml) and in flasks versus test tubes. It has been demonstrated that the culture volume and type of vessel used affect the dynamics of survival and therefore the selective pressures that populations experience (41).

Despite the differences in experimental design, there are still mutated genes shared between these two studies and the work presented here. Mutant alleles of *cpdA*, *putP*, *proQ*, *crp*, *glpR*, *hfq*, *rpoS*, *sdhB*, and *rimJ* were identified as core mutations here and in at least one of the other studies, further demonstrating their relevance for survival in an LTSP environment. Mutations in the global regulator genes observed here, such as *hfq* and *rpoS*, as well as in the genes encoding the core transcriptional machinery from these other studies, may point to global transcriptional dysregulation as an early strategy for adaptation during LTSP. Global dysregulation followed by fine-tuning to restore transcription to a prestress or new optimal state has been proposed as a strategy to adapt to stressful environments (42, 43).

We ultimately chose to sequence clones versus using a metagenomic approach because of the relatively low biomass obtainable from each time point sample (100 to 10,000 cells). Outgrowing frozen samples in fresh medium to obtain more biomass might have affected the relative frequency of genotypes in the population, particularly since clones from L2 exhibit a longer lag phase in fresh medium (Fig. 6B). The strategy of sequencing individual clones also allowed us to identify precise genotypes within the population, as well as the additional underlying variation, which we define as collateral genotypes. It is difficult to distinguish low-frequency bona fide mutations from sequencing errors using whole-population sequencing, and studies that have done so relied on temporal data; however, most collateral genotypes in our data are present at a single time point (44, 45). Even though most of these collateral genotypes were observed only once, they reflect the abundance of genetic diversity present within the culture during LTSP. Since only ∼48 cells were sampled (Fig. S6) from a large population (10^4^ to 10^6^ CFU/ml), variants must be present at a high enough frequency to be detected. Accordingly, the variants identified here likely reflect only the tip of the “genetic iceberg.” Nevertheless, they represent the most numerically dominant members of the population at that point in time.

The population dynamics of multiple coexisting subpopulations observed in the four parallel populations has been identified by other studies, including some of the earliest microbial evolution experiments (53, 54, 45–48). The fluctuations in population size during LTSP are believed to be caused by nutrient changes in the environment. After a specific nutrient is depleted, genotypes associated with its utilization decrease in frequency, while new mutants able to utilize a new energy source or capitalize on another existing nutrient increase in frequency. It is hypothesized that the rates of death and growth between these newer and older mutant populations are likely unequal, creating the fluctuations in total cell counts observed (Fig. 1). Within population 2, we detected two instances of a core genotype reverting back to the wild-type allele; the *hfq*(I44S) mutation in L2 and the acs mutation in L1 revert in a sublineage (Fig. 3A). We believe that these results further illustrate the dynamism of the LTSP chemical environment. Among the four parallel populations, we detected 38 alleles of *hfq* suggesting they were under strong positive selection, and reversion of the *hfq* allele in L2 suggests it was no longer beneficial in the context of its current environment.

The pool of available nutrients during LTSP is likely dynamic as cells adapt to recycling cellular waste and debris. It is not surprising that presumed beneficial mutations tended to be in genes either directly or indirectly involved in metabolism. These mutations likely improve the capacity to effectively utilize a nutrient, providing a competitive advantage. Mutations in transport proteins likely provide a fitness advantage by allowing cells to import nutrients from their environment and/or excrete waste products more efficiently.

Throughout the 1,200 days of incubation in population 2, the relative numbers of cells from L1 and L2 varied as L1 and L2 continued to acquire new mutations (Fig. 4). Despite the large fluctuations in their frequencies, including population numbers occasionally below our limit of detection, neither L1 nor L2 permanently dominated the population. It is likely that each occupied a specific niche(s), and their relative frequency fluctuated as their particular niche(s) expanded or contracted. Another possibility is that these fluctuations are caused by syntrophic interactions between L1 and L2, phenomena that have been observed in experimentally evolved microbial populations in constant environments, which would likely impose constraints on niche size based on the output of a public good (14–16). Differences in outgrowth and competitive phenotypes of L1 and L2 further support that these two lineages are physiologically distinct from one another (Fig. 6 and 7).

In contrast to L1 and L2, the genotypes in L3 and L4 are relatively invariant for the 150 days they are present, each fixing only one additional mutation after day 10 (Fig. 2). One explanation for the relative lack of mutational sweeps and genetic diversity exhibited by cells from L3 and L4 is that they are niche specialists, residing near the apex of a narrow fitness peak. The eventual disappearance of L3 and L4 from the population may be the result of the loss of such a particular niche: once the nutrients defining their niches were no longer present, their ability to compete well enough to remain detectable was lost (49).

Evolution experiments using yeast and E. coli cells that are serially passaged have shown mutations fixing in populations as mutational cohorts (44, 45). In our study, most genotypes are distinguished by a single mutation (Fig. 4), a difference that is potentially due to experimental design. In serial passage experiments, a small fraction of a dense culture is transferred to fresh medium daily, keeping the population essentially in exponential-phase growth. Daily bottlenecking causes the genotypes present at the highest frequency to be propagated in the population, meaning those cells are the fastest growing in the population, and along with fast growth comes more mutations. In our system, growth is relatively slower and likely happens in punctuated bursts as beneficial mutations arise in the population. Our system also has an apparent carrying capacity since the population size remains roughly stable, but based on the diversity and selective sweeps observed, substantial growth must also occur.

This work provides the most comprehensive analysis of the population structure for cells in a long-term batch culture to date, detailing the population dynamics in a complex ever-changing environment. We present evidence for both dynamic change and long-term stability (e.g., strong selective sweeps and the coexistence of two lineages for 1,200 days) and identify mutations that may be beneficial during LTSP. The changing nutritional environment during LTSP is more akin to what bacteria experience in real world environments, where cells spend a majority of the time under conditions of starvation and stress.

## MATERIALS AND METHODS

### Incubation of long-term cultures.

All cultures were grown in in 5.0 ml of lysogeny broth/Luria-Bertani broth (LB) in an 18- by 150-mm borosilicate test tube and incubated at 37°C with aeration in a humidified warm room (65 to 70% relative humidity) in a TC-7 roller drum (New Brunswick Scientific, Edison, NJ). The long-term evolution cultures were inoculated with a clone of E. coli K-12 W3110 lineage strain ZK1142 or ZK1143 (7) from a frozen glycerol stock and incubated for 1,200 days. Ten microliters of the culture was saved on days 10, 20, and 30 and every subsequent 30 days in LB plus 10% glycerol and frozen. Starting at day 30, prior to each sampling, sterile distilled water was added to the culture to restore volume to 5.0 ml (with typically 0.25 to 0.5 ml of water added [data not shown]). The number of CFU/ml was obtained by determining the titer of a sample of the culture on LB agar plates. Cell counts via microscopy were performed on three occasions and corresponded to the CFU determined cell counts (data not shown).

### Whole-genome resequencing of clones.

Clones were isolated by spreading samples from the frozen glycerol stocks of each saved population onto LB agar plates at sufficient dilution to yield individual colonies. Clones from day 240, while forming colonies, did not grow well in liquid LB medium making it difficult to isolate DNA for sequencing libraries. Therefore, we were only able to sequence 15 clones from day 240. After overnight growth at 37°C, single colonies were picked and grown in 800 μl of LB in individual wells of a 96-well plate and incubated for 48 h at 37°C. DNA was extracted from 96-well plate cultures using Qiagen DNeasy 96 blood and tissue kits. Whole-genome sequencing libraries were prepared using the Illumina Nextera kit, where each clone was labeled with two unique barcodes. Equimolar amounts of ∼192 libraries or ∼4 time points were pooled and run on an Illumina NextSeq or HiSeq lane. Sequencing was performed through the USC Sequencing Core and BGI, Davis, CA.

### Identification of mutations in clones.

Reads were aligned to the parental ZK126 reference genome (50), using Breseq version 0.32.1 (51) in consensus mode to identify SNPs, small indels, large deletions, and novel insertion elements (ISs). Only clones with an average of 20-fold read depth across the genome were included in further analyses. GenomeDiff files for each clone were analyzed using the gdtools compare function from Breseq to compile all mutations in all 1,117 clones. These mutations were then parsed to identify unique mutations, which may be present in one or more clones (Data Set S1). A presence-absence matrix of mutations present in each clone was then generated (Data Set S1). The parental strains, ZK1142 and ZK1143, have 11 mutations relative to the reference genome used from ZK126 at positions 2894668 (*yqeG*), 2499451 (*ypfM*/*yffB*), 1810671 (*yobD*/*mntP*), 1398837 (*ydcI*), 1576168 (*ynfM*/*asr*), 2166923 (*psuK*/*fruA*), 281996 (*frmR*/*yaiO*), 2246663 (gyrA), 2959436 (*ubiI*), 3457470 (*yihU*), and 4175949 (*ghxP*). Mutations present within an E14 cryptic prophage were removed from further analysis because the region was highly variable, and the reference sequence is lower quality in this region. Large amplifications were identified by plotting the coverage across the genome and visually locating regions with greater coverage (see Fig. S5 in the supplemental material).

### Phylogenetic organization.

To visualize phylogenetic relatedness, a minimum-spanning tree (MST) of the sequenced clones was generated using Phyloviz (52). Sequence types were produced by identifying unique genotypes among clones that were then placed into sequence type groups, and a full MST using the geoBURST algorithm was generated (Data Set S2). Sublineages were defined as branches diverging from “core genotypes” (genotypes resulting from mutations that fixed within a lineage) possessing 10 or more clones.

10.1128/mBio.03337-20.10 DATA SET S2 Sequence types and isolate metadata from culture 2 used to build minimum-spanning tree. Download Data Set S2, XLSX file, 0.7 MB. Copyright © 2021 Ratib et al. 2021 Ratib et al. This content is distributed under the terms of the Creative Commons Attribution 4.0 International license.

### Growth and physiology of population 2 isolates.

Isolates used for growth and competition assays are listed in Table S1. Overnight cultures were then subcultured by inoculating a dilution of cells into fresh medium. The growth and survival of cells were monitored by serial dilution of population samples into LB and plating onto LB agar plates. After incubating the plates overnight at 37°C, the number of colonies were enumerated for the dilution with the greatest number of countable colonies. The limit of detection for cell counts was 1,000 CFU/ml for growth experiments, unless otherwise stated.

### Competition assays.

Competed strains were grown in monoculture overnight at 37°C in 5 ml of LB. To initiate competition experiments, 5 μl of overnight cultures for strains to be competed was inoculated into the same test tube containing 5 ml LB medium. Strains were differentiated by determining the titers of strains onto LB plates with strain-specific antibiotics: streptomycin (25 μg/ml) or nalidixic acid (20 μg/ml).

### Data availability.

Raw sequencing reads have been deposited in the NCBI BioProject Database under accession number PRJNA687255 (https://www.ncbi.nlm.nih.gov/bioproject/687255).

10.1128/mBio.03337-20.7 FIG S6 Hierarchical clustering and random subsampling of genotypes from days 150 and 210. The dendrogram was generated from hierarchical clustering of genetic distance of clones from days 150 (A) and 210 (B). Colors correspond to genotype calls resulting from cutting the tree at k = 3. Scatterplots showing the number of genotype groups detected when randomly subsampling sequenced clones after cutting the tree at k = 2 to k = 5. Analysis is shown for days 150 (C) and 210 (D). Download FIG S6, PDF file, 0.2 MB. Copyright © 2021 Ratib et al. 2021 Ratib et al. This content is distributed under the terms of the Creative Commons Attribution 4.0 International license.
