# Supplementary figures and images for: Evolution in Long-Term Stationary-Phase Batch Culture: Emergence of Divergent Escherichia coli Lineages over 1,200 Days
Source: mBio. 2021 Jan 26;12(1):e03337-20. doi: 10.1128/mBio.03337-20 (PMC7858067; doi:10.1128/mBio.03337-20)

Frequency

0.4  
0.3  
0.2  
0.1  
0.0

A:T > G:C

G:C > A:T

A:T > T:A

A:T > C:G

G:C > T:A

G:C > C:G

Transitions

Transversions

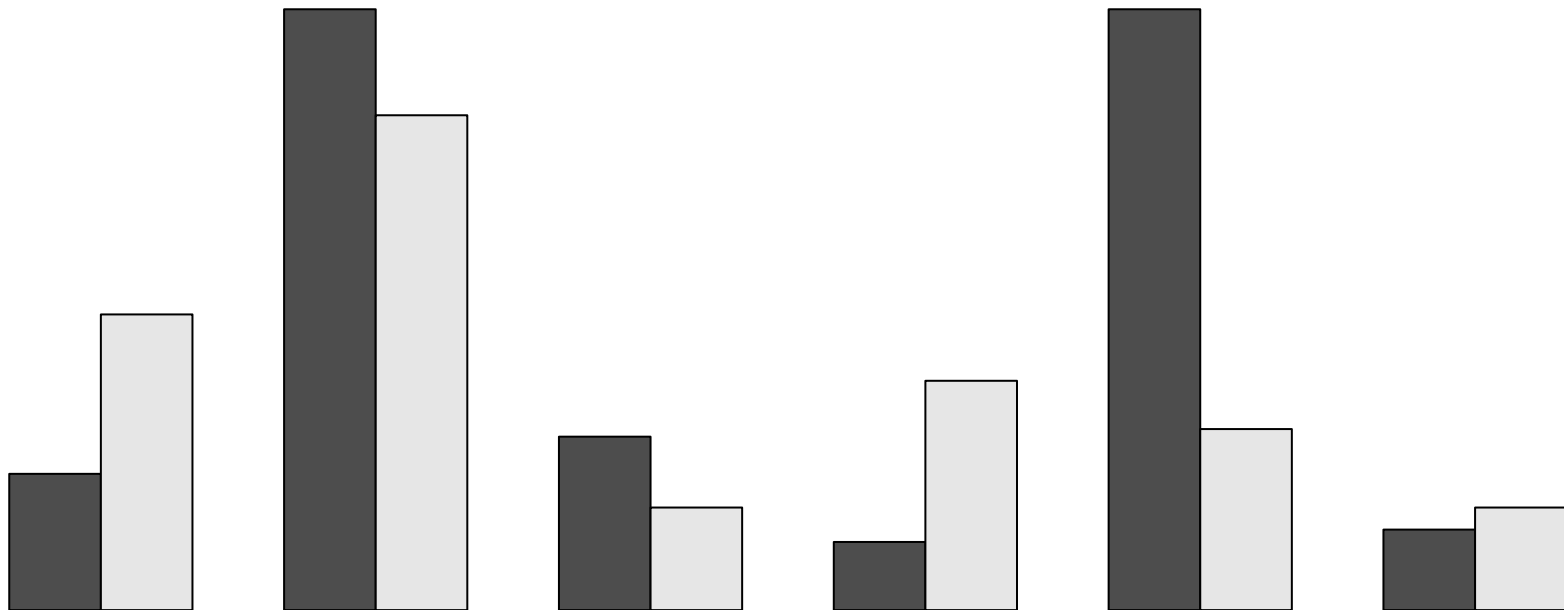

Supplement: FIG S1 [file mBio.03337-20-sf001.pdf]

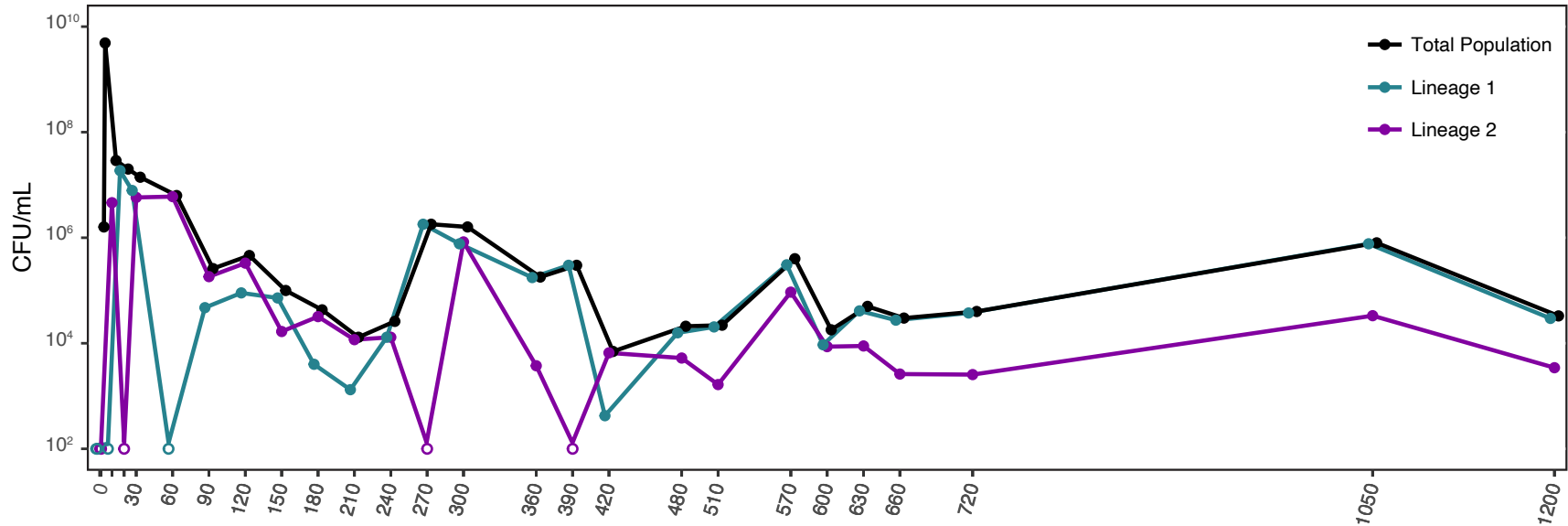

Supplement: FIG S2 [file mBio.03337-20-sf002.pdf]

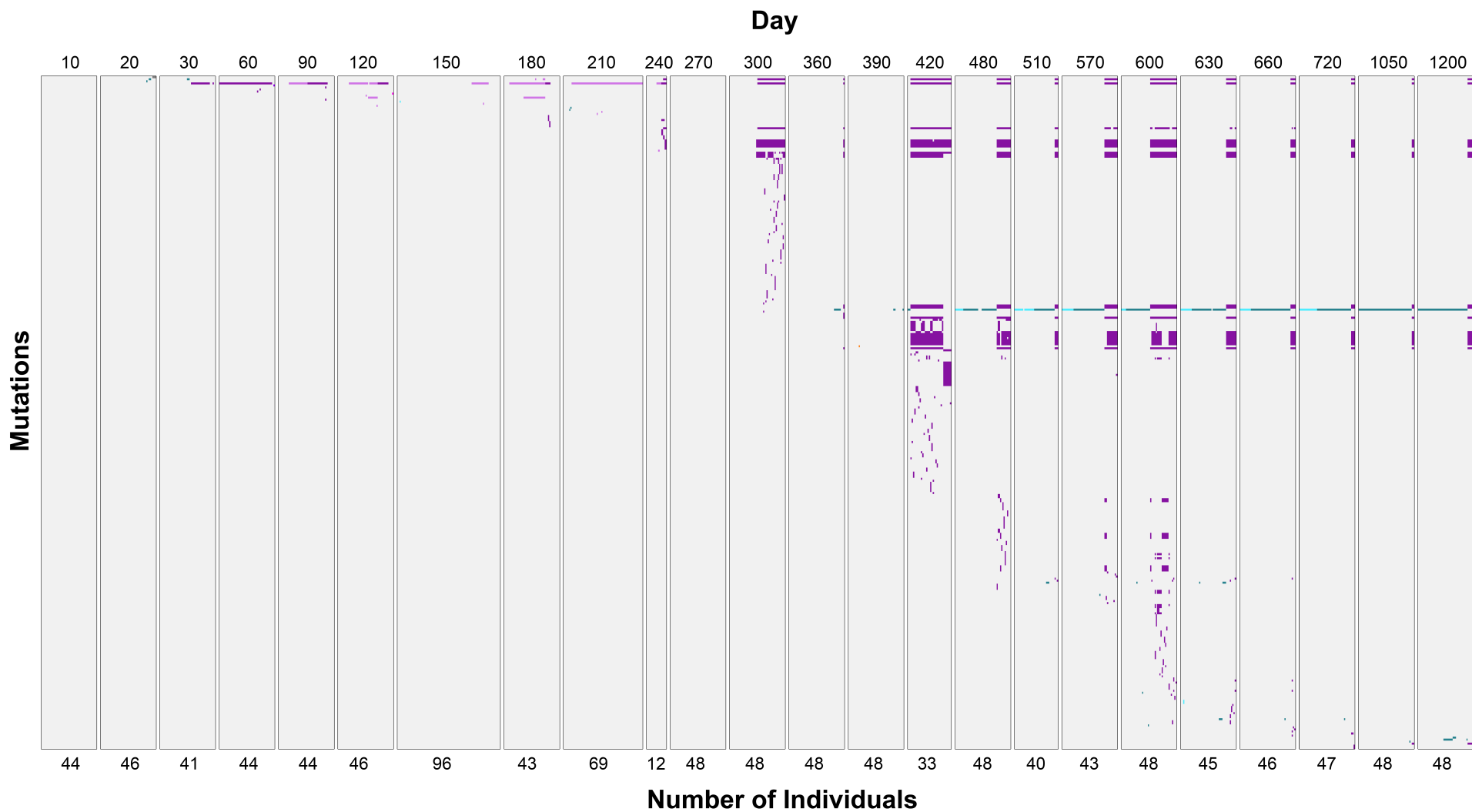

Supplement: FIG S3 [file mBio.03337-20-sf003.pdf]

*ntpA*

*rpoS*

Expression Fold Change (Log2)

32  
16  
8  
4  
2  
1  
0.5  
0.25  
0.125  
0.0625

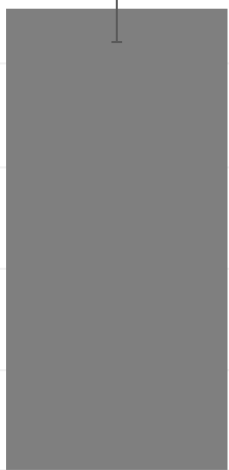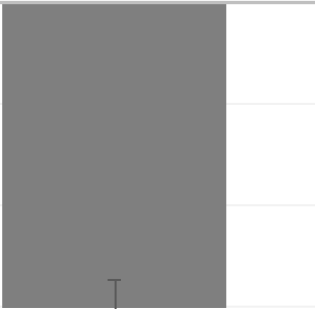

Supplement: FIG S4 [file mBio.03337-20-sf004.pdf]

**Day**

10

150

180

180

240

300

300

300

420

420

420

480

600

600

**Coverage/Mean Coverage**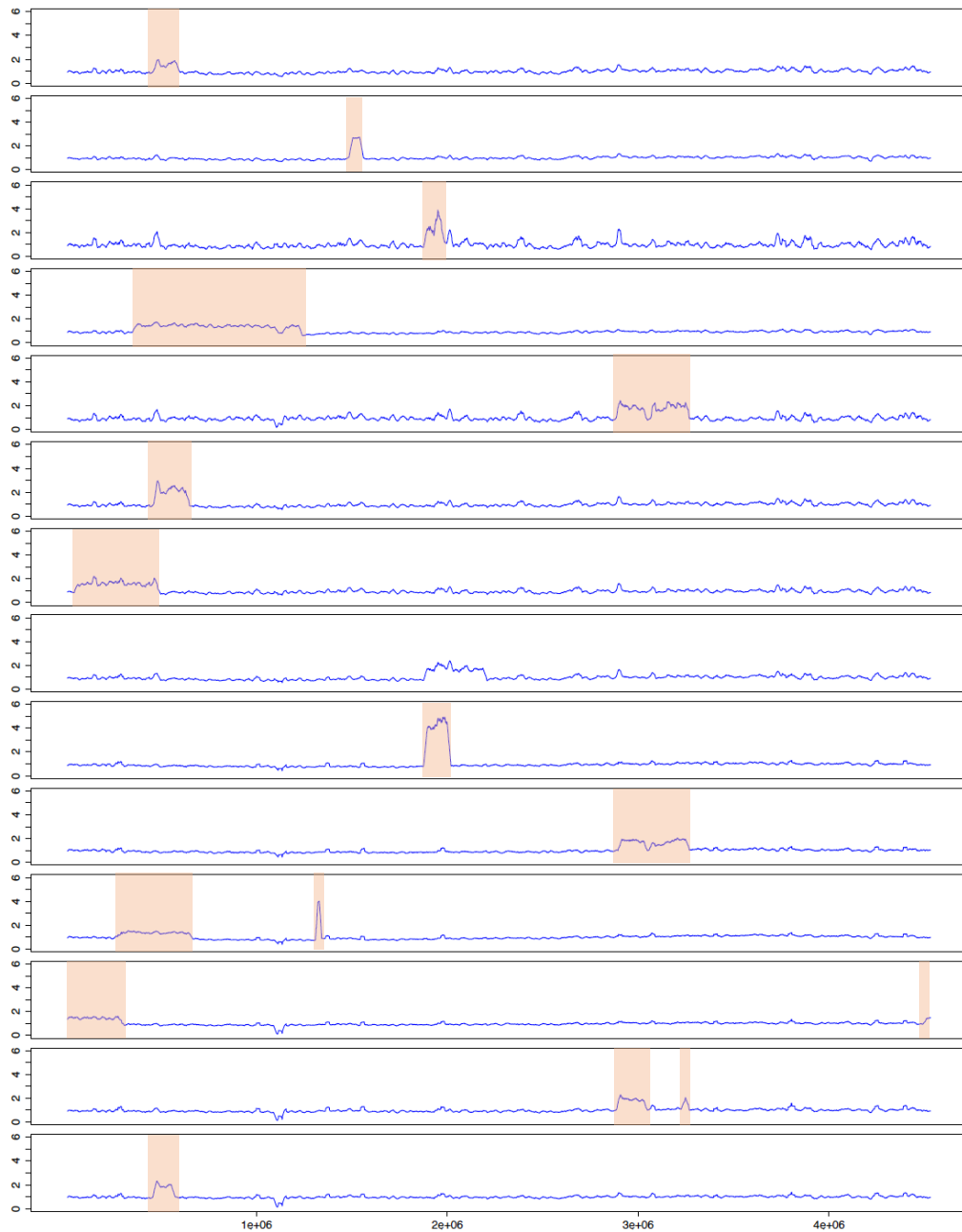**Genome Position**

Supplement: FIG S5 [file mBio.03337-20-sf005.pdf]

**A**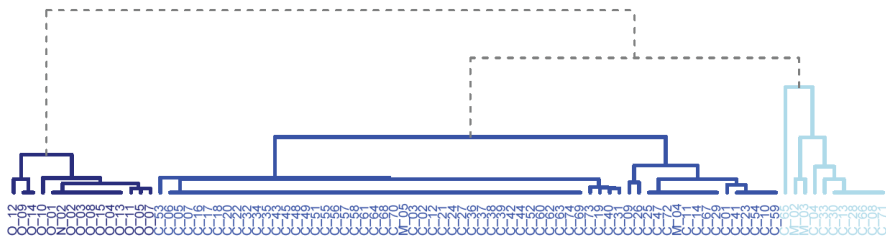**B**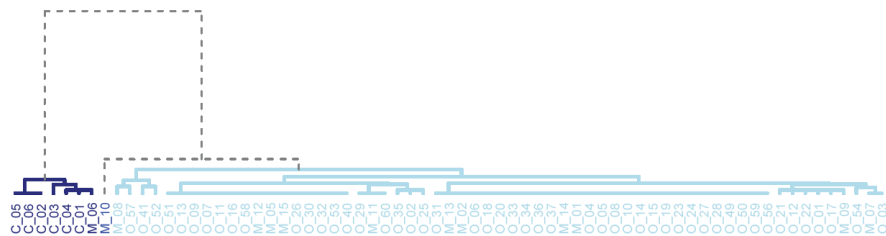**D**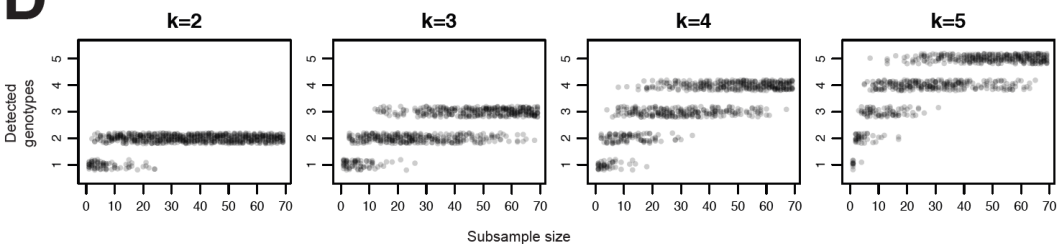**C**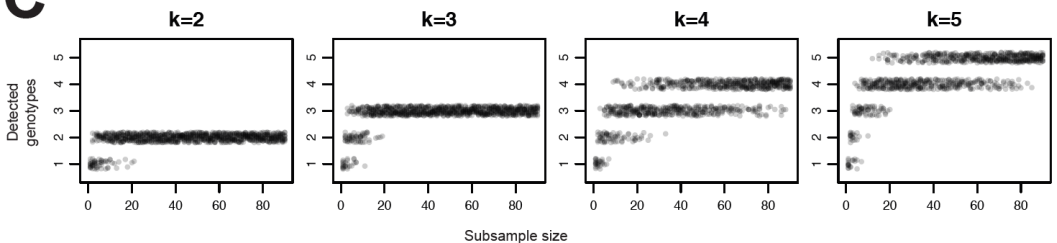

Supplement: FIG S6 [file mBio.03337-20-sf006.pdf]
