# Supplementary material for: Evolution in Long-Term Stationary-Phase Batch Culture: Emergence of Divergent Escherichia coli Lineages over 1,200 Days
Source: mBio. 2021 Jan 26;12(1):e03337-20. doi: 10.1128/mBio.03337-20 (PMC7858067; doi:10.1128/mBio.03337-20)
Supplement: TEXT S1 [file mBio.03337-20-s0001.pdf]

Measured expression of *dtpA* and *rpoS* using qRT-PCR. Three biological replicates of each strain (wild-type, *dtpA* mutant, and *rpoS* mutant) were grown in LB at 37°C for four hours then mRNA was isolated using the Qiagen RNeasy kit. cDNA was synthesized from the extracted mRNA using the Tetro cDNA Synthesis Kit from Bioline. Primer pair efficiency was determined using four ten-fold serial dilutions (1ng, 0.1ng, 0.01ng, and 0.001ng) of *E. coli* genomic DNA to construct a standard curve. To quantify gene expression, 2ng of cDNA was mixed with 10uM of each primer in a 20uL reaction volume and tested using the CFX96 Real Time PCR Detection System (Bio-Rad) and SYBR Green SensiMix (Bioline). Cycle threshold (Ct) values were then compared between wild-type and the mutants to calculate relative gene expression using the Pfaffl method (53). Gene expression was also normalized to *idnT* as a reference gene (54).

Glycogen and catalase assays to measure RpoS activity. Clones with mutations in *hfq* or *rpoS* were grown to stationary phase in LB broth. 10µL of stationary phase cells were then spotted onto LB plates and incubated overnight at 37ºC to produce spot lawns. These spot lawns were either flooded with concentrated iodine to test the amount of glycogen (55) present or 20 µL hydrogen peroxide was dropped onto a spot lawn to test the amount of catalase (36) present in cells.

Subsampling to determine number of clones to sequence. To estimate the population diversity we performed an initial sequencing of 96 clones from day 150 and 69 clones from day 210. We identified variants in these clones by the process described below and calculated a genetic distance matrix with the R function dist(). We used the tree.cut() function to cut the tree at k=2,3,4,5 and assigned genotype calls to each clone. We then performed random subsampling without replacement of these genotypes in steps of n=1 from 1 to the number of clones sequenced. We chose to sequence 48 clones per time point (Fig. S5) as most genotypes were recovered when 60

and 40 clones were subsampled for days 150 and 210, respectively. We reasoned that with 48 clones we would capture the major genotypic clades present and if a time point appeared particularly diverse, we could easily sequence more clones from the frozen samples.
