# Supplementary material for: Evolution in Long-Term Stationary-Phase Batch Culture: Emergence of Divergent Escherichia coli Lineages over 1,200 Days
Source: mBio. 2021 Jan 26;12(1):e03337-20. doi: 10.1128/mBio.03337-20 (PMC7858067; doi:10.1128/mBio.03337-20)
Supplement: Supplementary file 4 [file mBio.03337-20-st001.docx]

Supplementary Table 1 | Isolates used for growth and competition experiments

Strain	Origin	Relevant Genotype	Relevant Phenotype	Reference
ZK1142	ZK126	Nal^r		Zambrano *et al*. (1996)
ZK1143	ZK126	Str^r		Zambrano *et al*. (1996)
L1.1	ZK1142	*dtpA* -108 A$\rightarrow$T [b], *rpoS* -573 A$\rightarrow$G [b], *sdhB* M107I [a]	*dtpA* mutation results in increased expression. *rpoS* mutation results in decreased expression	This study
L1.2	L1.1	*cpdA* S222L [a], *glpR* N91fs [a]		This study
L1.3	L1.2	*rimJ* A61fs [a]		This study
L1.4	L1.3	*metB* P146S [a]		This study
L2.1	ZK1142	*hfq* I44S [a]		This study
L2.2	L2.1	*paaX* R74C [a], *lrp* IS2 insertion, *rne* G174D [a]		This study
L2.3	L2.2	*proQ* R80C [a], *insH1* 1bp deletion [b]		This study
L2.4	L2.3	*clpX/lon* IS186 insertion, *putP* A337T [a]		This study

[a] Mutation resulting in amino acid change. [b] Intergenic mutation affecting promoter.
